# Factors Associated with Physician Agreement and Coding Choices of Cause of Death Using Verbal Autopsies for 1130 Maternal Deaths in India

**DOI:** 10.1371/journal.pone.0033075

**Published:** 2012-03-28

**Authors:** Ann L. Montgomery, Shaun K. Morris, Diego G. Bassani, Rajesh Kumar, Raju Jotkar, Prabhat Jha

**Affiliations:** 1 Centre for Global Health Research, Li Ka Shing Knowledge Institute, St. Michael Hospital, Toronto, Ontario, Canada; 2 Institute of Medical Science, University of Toronto, Toronto, Ontario, Canada; 3 Division of Infectious Diseases, Hospital for Sick Children, Department of Pediatrics, University of Toronto, Toronto, Ontario, Canada; 4 Dalla Lana School of Public Health, University of Toronto, Toronto, Ontario, Canada; 5 School of Public Health, Post Graduate Institute of Medical Education, Chandigarh, India; Aga Khan University, Pakistan

## Abstract

**Background:**

The Indian Sample Registration System (SRS) with verbal autopsy methods provides estimations of cause specific mortality for maternal deaths, where the majority of deaths occur at home, unregistered. We aim to examine factors that influence physician agreement and coding choices in assigning causes of death from verbal autopsies.

**Methodology/Principal Findings:**

Among adult deaths identified in the SRS, pregnancy-related deaths recorded in 2001–2003 were assigned ICD-10 codes by two independent physicians. Inter-rater reliability was estimated using Landis Koch Kappa classification 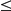 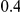
– poor to fair agreement; >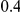 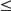 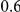
– moderate agreement; >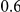 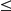 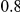
– substantial agreement; >
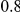
– high agreement. We identified factors associated with physician agreement using multivariate logistic regression. A central consensus panel reviewed cases for errors and reclassified as needed based on 2011 ICD-10 coding guidelines. Of 1130 pregnancy-related deaths, 1040 were assigned ICD-10 codes by two physicians. We found substantial agreement regardless of the woman's residence, whether the death was registered, religion, respondent's or deceased's education, age, hospital admission or gestational age. Physician agreement was not influenced by the above variables, with the exception of greater agreement in cases where the respondent did not live with the deceased, or early gestational age at the time of death. A central consensus panel reviewed all cases and recoded 10% of cases due to insufficient use of information in the verbal autopsy by the coding physicians and rationale for this reclassification are discussed.

**Conclusion:**

In the absence of complete vital registration and universal healthcare services, physician coded verbal autopsies continues to be heavily relied upon to ascertain pregnancy-related death. From this study, two independent physicians had good inter-rater reliability for assigning pregnancy-related causes of death in a nationally-represented sample, and physician coding does not appear to be heavily influenced by case characteristics or demographics.

## Introduction

One-fifth of the global maternal deaths occur in India [1]. The United Nation's Millennium Development Goal Number 5 targets a 75% reduction in the maternal mortality ratio by 2015 through Safe Motherhood Initiatives [2]. However, maternal deaths are relatively rare events that are prone to under-reporting and misclassification, particularly in countries lacking a comprehensive vital registration system, and in which the majority of deaths are unregistered and occur outside the healthcare system [3], [4]. Robust regional, national, and global estimates of cause-specific maternal mortality are essential in generating political will around the issue, for monitoring trends, and directing and justifying investments in effective programs [5]–[8].

Two methods have been developed for deriving cause of death codes from verbal autopsy interviews with family respondents; physician review, and computer automated methods [9]. Physician review relies on one or two physicians reviewing information from the closed-questions and/or transcribed narrative in the verbal autopsy followed by assignment of cause of death code.

Physician coding of cause of death mimics the scenario physicians use when taking a history in a clinical setting. Physicians consider the deceased's reported health history, demographic factors in the case, the temporal relationship of the appearance of symptoms with the suspected clinical condition, as well as the reported symptoms themselves in the typical presentation of a case. There have been a number of efforts to standardize and validate physician cause of death assignment [10]–[13]. However, the challenge of estimating the validity of physician cause of death assignment in all VA studies is that *a priori* verification of cause of death requires the same functioning high quality healthcare system that was often lacking at the time of individual's death.

This study is limited to examining what factors influence physician coding by estimating the inter-rater agreement of two physician coded cause of maternal deaths from verbal autopsies. This study also examines where physicians err with respect to established guidelines.

## Methods

The Indian Million Death Study (MDS) is being conducted by the Registrar General of India in collaboration with the Centre for Global Health Research at the University of Toronto. The MDS uses the Sample Registration System (SRS) to monitor cause-specific mortality in the population. Details of the study are explained in detail elsewhere [14] and are summarized here. This study uses data collected in 2001–2003. An average of 150 households were drawn from 6671 randomly selected sample units in all 28 states and 7 union territories. Every birth and death in the home were independently recorded during monthly visits by trained non-medical enumerators and every six months by Registrar General of India (RGI) surveyors [13], [15]–[17].

RGI interviewers collected details about the events that preceded the deaths using a validated verbal autopsy tool called RHIME (Routine, Reliable, Representative, and Re-sampled Household Investigation of Mortality with Medical Evaluation) consisting of responses to structured questions and an open-ended narrative provided by the respondent, in the respondent's own language. For all deaths of women 15–49 years, interviewers asked respondents specifically whether the woman was pregnant or 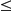
42 days post-abortion/miscarriage/partum. A specific maternal death questionnaire and verbal autopsy RHIME was completed for all of these cases [17].

Physicians (referred to as ‘coding physicians’) were trained in the International Classification of Disease 10th edition (ICD-10) cause of death assignment by the Million Death Study team. Two physicians independently reviewed the questionnaires and verbal autopsy narratives, in one of 15 languages, and assigned a cause of death using ICD-10 [18], [19].

World Health Organization definitions were used (Table 1) [1]. ICD-10 codes were grouped into the traditional cause of pregnancy-related death categories (Table 2). Verbal autopsies were translated into English.

**Table 1:** Definition of pregnancy-related deaths [33].

Pregnancy-related death	death of a woman during the pregnancy or 42 days postpartum regardless of cause
Direct maternal death	death of a woman during pregnancy or 42 days postpartum due to obstetric complications or its management, includes postpartum suicide
Indirect maternal death	death of woman during pregnancy or 42 days postpartum due to disease other than obstetric complications
Maternal death	includes direct and indirect maternal deaths
Injury-related deaths	death during pregnancy and up to 42 days postpartum due to violence, accidental death, or antenatal suicide

**Table 2:** Categorization of ICD-10 for pregnancy-related deaths.

	Category	ICD-10 Code
1	Obstetric hemorrhage	O44, O45, O46, O67, O71, O72
2	Maternal sepsis	O23, O41, O85, O86, A34
3	Hypertensive disorders of pregnancy	O10–O16
4	Abortion or Miscarriage	O03–O07, O20
5	Other obstetric conditions	O00, O21, O22, O26, O29, O74, O75, O87–O90, O95 &F53
6	Obstructed labour	O64–O66
7	Indirect obstetric death	O98–O99, A00-N99, R99
8	Injury	S00-Y09, Y60, Y69*

* Y60 & Y69 are classified as ‘Other obstetric conditions’ if these unanticipated surgical complications occur during labour and delivery.

Details about the death were coded by a panel of two physicians (SKM and PJ) and a midwife (ALM), referred to as the ‘consensus panel’, using 2011 ICD-10 coding guidelines (Web Appendix S1) and a validated coding tool called the Maternal Date Extraction Tool (M-DET) [20], both developed for maternal death verbal autopsies. The consensus panel received simultaneous training for approximately three hours on ICD-10 coding and the M-DET. The consensus panel was directed to independently review MDS physician coded deaths for further quality assurance, the short answer responses and narratives. Coders were instructed to take the narrative as the standard if there was a contradiction between the short answer response and the narrative. The consensus panel would reassign the physician coded cause of death code provided that (a) the physician had insufficiently used information provided in the verbal autopsy to meet the definition for the ICD-10 code of cause of death or (b) the 2011 ICD-10 coding guidelines provided direction to reclassify the case either because of an expanded case definition or more specific definition of onset of symptoms relative to delivery.

We investigated mechanism of item-nonresponse data using 2×2 Chi squared tables to determine the association of missingness with the outcome, physician agreement, in order to determine whether missing at random could be assumed. We used multiple imputation by chained equation to generate plausible values of item-nonresponse [21].

Proportional characteristics of cases, unweighted for survey data, were calculated using the imputed dataset. Inter-rater agreement between coding physicians was estimated using unweighted kappa statistic for nominal categorical variables, and 95% confidence intervals were calculated using bootstrap estimation of standard error for kappa method [22]. The Landis and Koch classification of inter-rater reliability was used to interpret the coefficients kappa: 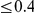
– poor to fair; >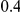 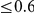
– moderate agreement; >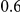 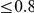
– substantial agreement; >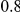 – high agreement [23].

We conducted 2×2 Chi squared univariate analysis to identify demographic, socioeconomic, geographic and individual factors that could influence physician agreement. We then conducted a multivariate logistic regression model to determine the association of physician agreement with those univariate covariates significant to a p-value of 0.2. We considered those covariates as significantly associated with physician agreement if the p-value was 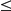
0.05. States of low (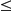
125 per 100 000 livebirth), medium (>126 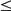
254 per 100 000) and high maternal mortality ratio (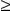
375 per 100 000) were generated from the Registrar General of India 2004–06 estimates [24]. Hospital admission refers to any inpatient admission for routine delivery or emergency admission.

All analysis was conducted using Stata version 11 (StataCorp. 2009. Stata Statistical Software: Release 11. College Station, TX: StataCorp LP).

SRS enrolment is on a voluntary basis, and its confidentiality and consent procedures are defined as part of the *Registration of Births and Deaths Act, 1969*. Verbal consent was obtained in the first SRS sample frame. The new SRS sample obtains written consent at the baseline. Families are free to withdraw from the study. The study poses no or minimal risks to enrolled subjects. All personal identifiers present in the raw data are anonymized before analysis. The MDS study using this SRS data has been approved by the review boards of the Postgraduate Institute of Medical Education and Research, Chandigarh, India and St. Michaels Hospital, Toronto, Canada.

## Results

### The study population

In 2001–2003, there were a total of 1130 pregnancy-related deaths among 10 069 deaths of all women aged 15 to 49 years in the MDS sample. Deaths were excluded from the analysis when no ICD-10 codes were assigned by the coding physicians (n = 90). Both physicians agreed on the categorization of 752 deaths (72.3%) at the first coding stage. The remaining 288 cases underwent a process of reconciliation in which the initial ICD-10 codes and the keywords assigned by each physician were exchanged between the two physician coders and an agreement was achieved in another 133 cases (12.8%). The remaining 155 cases (14.9%) required adjudication by a third, senior physician who reviewed the codes and assigned a final ICD-10 code (Figure S1).

Data were complete for 37% of the cases, and missing 1–2 values for 48% of the cases. Nine per cent of cases were missing 3 values, and the remaining 6% of cases were missing 4–11 values in the dataset. The data are assumed to be missing at random since the item-missing data mechanism does not appear to depend on the unobserved values. This was illustrated using 2×2 Chi-squared test of physician agreement (the outcome) on the missingness of the variable was found to be not significant (p-value
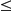
0.05) for all but one variable, term pregnancy (p = 0.03). Given the multiple comparisons, using the Bonferroni correction, there is likely no significant association. Imputed data had equal fraction of missing information (<0.2), an assumption necessary for variable inclusion in the logistic regression analysis.

The majority of the pregnancy-related deaths were in Indian states known as Empowered Action Group and Assam (states with high fertility and low income), in rural areas, and two-thirds of families reported that they did not register the woman's death (Table 3). The majority of women were between the ages of 20–29 years, at term pregnancy, and half reported hospital admission for labour or medical complication.

**Table 3:** Proportion of population of characteristics and unweighted kappa for physician agreement of cause of death categorization, by covariate.

Variable		n	(%)	kappa	(95%CI)	
India	Overall	1040	100.0	0.66	0.63	0.70
MMR by region*	Low	85	8.2	0.69	0.48	0.78
	Medium	266	25.6	0.63	0.57	0.69
	High	689	66.2	0.67	0.63	0.71
Rurality	Rural	938	90.2	0.67	0.64	0.72
	Urban	102	9.8	0.59	0.47	0.71
Death Registration	Yes	396	38.1	0.69	0.63	0.75
	No	644	61.9	0.68	0.64	0.73
Relationship with respondent	Husband or mother-in-law	341	32.8	0.65	0.61	0.71
	Other	699	67.2	0.67	0.62	0.71
Respondent lived with deceased	Yes	806	77.5	0.63	0.59	0.68
	No	234	22.5	0.74	0.67	0.80
Religion	Hindu	814	78.2	0.66	0.63	0.70
	Muslim	161	15.5	0.67	0.58	0.74
	Other	65	6.3	0.60	0.46	0.71
Education of deceased	Illiterate	636	61.2	0.65	0.60	0.68
	Literate	404	38.8	0.68	0.61	0.73
Education of respondent	Illiterate	468	45.0	0.64	0.59	0.69
	Literate	572	55.0	0.69	0.64	0.73
Occupation of deceased	Nonworker	696	67.0	0.64	0.59	0.68
	Worker	344	33.0	0.71	0.65	0.76
Gestational age	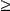 7 months	858	82.5	0.62	0.57	0.66
	<7 months	182	17.5	0.67	0.58	0.73
Hospital admission	Yes	472	45.4	0.66	0.58	0.71
	No	568	54.6	0.66	0.62	0.71
Maternal age	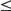 19 years	115	11.0	0.70	0.61	0.82
	20–29 years	539	51.8	0.63	0.56	0.67
	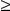 30 years	386	37.2	0.64	0.57	0.69

* Maternal mortality ratio by state of low (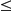
125 per 100 000 livebirth), medium (>126 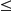
254 per 100 000) and high (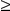
375 per 100 000) [24].

### Inter-rater agreement

Overall agreement between two coding physicians was substantial (Kappa = 0.66, 95% CI 0.63–0.70). Agreement was not influenced by place of residence, relationship of respondent to the deceased, whether the respondent lived with the deceased, respondent's literacy or deceased's literacy level, or deceased's occupation. Variables related to the pregnancy did not influence agreement: gestational age, and hospital admission (Table 3).

### Association of coder physician agreement with covariates

In the univariate analysis, there was no significant association between physician agreement and level of regional maternal mortality ratio (low, medium, or high), literacy level of the respondent, relationship of the respondent to the deceased (husband/mother-in-law versus other), hospital admission, whether or not the deceased received antenatal care in the pregnancy, or whether the death was registered. There was slightly greater physician agreement if the woman died when the pregnancy was at an early gestational age (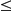
6 months gestation) (OR = 1.49, 95%CI 1.00–2.27, p-value 0.05) and when the respondent did not live with the deceased (OR = 1.47, 95%CI 1.01–2.13), when controlled for urban/rural categorization and language. A sub-analysis, stratified by those respondent who did and did not live with the deceased, and the physician coded category of cause of death did not result in greater agreement for ‘other obstetric cause’ (i.e. living with the deceased did not appear to be associated with a more specific cause of death code, conversely, not living with the deceased did not appear to be associated with a more non-specific cause of death code).

### Reclassification by consensus panel

Of the 1040 physician coded cases, there was agreement on the classification for two-thirds of cases by the consensus panel and one-third of cases were reclassified (the counts, unweighted for survey design, are summarized in Table 4) based on corrections of errors and further developed 2011 ICD-10 coding guidelines for maternal death verbal autopsy criteria (Web Appendix S1).

**Table 4:** Unweighted counts in agreement in cause of death assignment between physician coders and consensus panel of 1040 pregnancy-related deaths.

	Consensus Panel							
Physician Coders	Hemorrhage	Sepsis	HDP*	TA/SA**	Other+	Indirect	Injury	Obstructed++
Hemorrhage	**227**	43	18	8	35	11	0	0
Sepsis	5	**98**	0	4	2	5	0	0
HDP*	2	2	**30**	1	7	4	0	0
TA/SA**	0	0	1	**86**	4	1	0	0
Other+	18	15	16	4	**124**	16	22	0
Indirect	6	11	8	3	19	**109**	0	0
Injury	0	1	1	1	0	0	**32**	0
Obstructed++	19	3	1	0	34	5	0	0

* Hypertensive disorders of pregnancy.

** Complications from therapeutic abortion (TA) or spontaneous miscarriage (SA).

+ Other obstetric.

++ Obstructed labour.

For one-third of reclassified cases (n = 111, approximately 10% of all physician-coded cases), the physician coders could have better used existing information from the verbal autopsy. Of these, in 14 cases, ICD-10 codes were assigned to cases not meeting the gestational age definition, 20 cases were erroneously classified as late maternal deaths and 1 case was classified as a paediatric case. Within the septic cases, there were 8 cases in which the physician assigned a very general cause of death (i.e. O98, O99, R99) that were reassigned to obstetric tetanus cases and 68 cases were reclassified to intrapartum/postpartum sepsis (O41, O85 or O86). Most postpartum sepsis cases presented >3 days postpartum (50% of whom had died by day 7 postpartum, and 75% of whom had died by day 14 postpartum) with new onset of fever and/or abdominal pain, with any additional history or symptoms of prolonged rupture of membranes, prolonged labour, foul-smelling vaginal discharge or new onset of jaundice postpartum. This under-classification of maternal sepsis has been reported in other maternal death studies [25], [26].

The remaining 223 reclassified cases were attributed to expanded 2011 ICD-10 coding guidelines. In summary 27% of cases were reclassified obstructed labour cases, 30% of cases were reclassified from specific to the general category of ‘other obstetric’ due to insufficient information to meet the 2011 ICD-10 coding guidelines, 18% of cases were reclassified to non-obstetric causes as the 2011 ICD-10 coding guidelines which provides more specific criteria for onset of symptoms relative to delivery, and 10% of cases were reclassified to new categories not provided in the 2005 ICD-10 coding guidelines.

While obstructed labour is often cited in the research literature as a cause of maternal death, it is not a mutually exclusive category but rather a contributory condition leading to death by either obstetric hemorrhage (including uterine rupture), maternal sepsis or other unknown obstetric cause. The consensus panel reclassified the 62 cases to these three categories for all but 6 cases, which were reclassified to either hypertensive disorders of pregnancy (HDP) or indirect maternal death. Similarly, anaemia is considered a contributory conditions, and the 2005 ICD-10 coding guidelines direct physicians to code the under-lying pathophysiological cause of death, e.g. malaria, and to include anaemia in only the keyword section of the coding.

Sixty-eight women were reclassified to ‘other obstetric’; 47 from specific categories (obstetric hemorrhage, maternal sepsis, HDP, complications from abortion or miscarriage) due to insufficient information to meet the criteria of the 2011 ICD-10 coding guidelines; of these, 4 women were reclassified from HDP or hemorrhage due to venous complications in pregnancy or postpartum (O87, O88). There were 41 women with pre-existing or prolonged illness or illness arising in early in the pregnancy or late in the postpartum period, where the informant reported signs and symptoms of fever and/or jaundice, malaise, anorexia; or diagnosed malaria, hepatitis, tuberculosis, or cancer; and these women were reclassified as indirect maternal deaths. Twenty-three cases were reclassified to intrapartum sepsis (O41), a newly defined 2011 ICD-10 coding guidelines.

## Discussion

The aim of this study was to investigate the factors associated with physician agreement in the causes of pregnancy-related deaths based on verbal autopsy, and patterns of coding disagreement with a consensus panel. It was not designed to estimate the cause of death distribution, nor the validity of the cause of death assignment.

A large proportion of the women who died were never admitted to a health facility, nor were their deaths registered. It is within this context that verbal autopsies are of greatest benefit to estimate causes of pregnancy-related deaths. We found that other than gestational age and whether the respondent lived with the deceased, none of the variables studied influenced physician agreement in arriving at a cause of death. Lozano *et al.* compared physician cause of death assignment from VAs to a medically certified dataset of pregnancy-related deaths (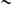
200 urban hospital deaths of two states in India) [27]. In this dataset, verbal autopsies were collected by interviewing respondents in cases where care was provided by professional healthcare providers. They found that physicians could assign an accurate diagnosis to a slightly higher proportion (65%) of cases when provided with the respondent's report of healthcare contact compared to VAs stripped of this information (57%). The study assumes that VAs stripped of all reference to healthcare contact were considered suitable proxies for cases who received no professional healthcare, and since all cases received professional healthcare in the medically certified dataset, respondents, who did not provide care for the deceased, were assumed to be sufficiently knowledgable and appropriate informants. In our study, there was no difference in inter-rater physician agreement for cases where hospital admission did or did not occur (K = 0.66), suggesting that the respondent's reporting of hospital admission, and the resulting professional care and communication, does not influence the physician coding choices.

We did not find variation in physician agreement when comparing deaths that came from regions of low versus high maternal mortality, nor was there higher agreement for women from urban areas compared to rural areas or between registered and unregistered deaths. Physician agreement does not appear to be influenced by whether the respondent was the deceased woman's husband or mother-in-law, both considered proxies for the main decision-maker with respect to maternal healthcare uptake [28], [29] compared with other respondents. As well, agreement was not influenced by literacy of the respondent. Access to services is higher in urban areas among the literate; therefore we hypothesized that the quality of narrative would be better when the respondent was literate, from an urban area, when there had been a hospital admission and where the death was registered. Similarly, neonatal and childhood death studies from the Million Death Study found no association of state, rural/urban, or death registration with improved physician agreement [30]. We found slightly greater agreement in cases where the gestational age of the pregnancy at the time of the woman's death was 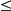
6 months. This is expected as an obstetric death at this time has, according to our categorization, only one cause - complication from abortion or miscarriage, versus term pregnancies (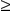
7 months gestation) can be categorized into either of four groups: obstetric hemorrhage, obstetric sepsis and tetanus, hypertensive disorders of pregnancy, and ‘other obstetric complication’. The slightly greater physician agreement noted when the respondent did not live with the deceased was surprising. We considered this may be related to a poor quality of narrative leading to greater agreement in coding of the non-specific category of ‘other obstetric complication’; however, we did not in fact find greater physician agreement in the sub-category category of ‘other obstetric condition’. While the husband or mother-in-law tends to be the primary decision maker for pregnant women in India, many members of the family or neighbourhood may care for the women in labour or in an emergency, resulting in perhaps a broader group of suitable respondents in the case of maternal death [31]. This may minimize the importance of who the respondent is, but rather whether the respondent cared for the woman around the time of death.

### Agreement between physician coders and consensus panel

In this study, physician coders are responsible for coding all newborn, child and adult deaths. Review of all pregnancy-related death cases by a small consensus panel brought further consistency in coding ICD-10 cause of death. As well, criteria for verbal autopsy has been further developed since the initial physician coding took place for these cases in 2005, therefore this review will provide comparable data from 2001 through to the present. The consensus panel applied 2011 ICD-10 coding guidelines (Web Appendix S1) which lead to reclassification of one-third of cases.

It was found that <10% of cases were reclassified due to coder error, where existing criteria was not adhered to (gestational age, postpartum time cut-off, signs of maternal sepsis or obstetric tetanus); whereas, the remaining cases were reclassified by the consensus panel for reasons of expanded criteria. Ongoing training for physician coders will address the more common mistakes in coding in addition to providing training in the expanded list of pregnancy-related cause of death criteria.

### Limitations

Because the two coding physicians may have different clinical training and experience, their ability to interpret and code a cause of death from the verbal autopsy may differ. However, due to lack of physician identifiers in the dataset, it was not possible to take account of individual physician characteristics in this analysis.

The selection of pregnancy-related deaths from the pool of all-cause female deaths relies on the respondent's knowledge and acknowledgement of the pregnancy. In early termination (either spontaneous or therapeutic), or in pregnancies of unmarried women, there is a selection bias due to the under-reporting of these groups.

We examined eight categories of cause of death, and the choice of categorization of the ICD-10 codes (Table 2) is based on traditional presentation of maternal reproductive complications in the literature. Such categorization is mutually exclusive, exhaustive, and sufficiently specific to inform public health policy. These categories limit the possibility of analysis of more specific cause of death, even though we understand that more detailed analysis of causes of maternal deaths are also a limitation of verbal autopsy studies due to the lack of medical records, clinical assessments, and laboratory findings.

There are 5% resampled verbal autopsy interviews for all-cause mortality as per the study protocol however, this sample frame was not stratified for cause of death, and resulted in <1% of re-interviewing of respondents in cases of pregnancy-related deaths. Therefore, we were unable to evaluate the study methodology and the repeatability of the interview process and physician coding to illicit the same cause of death assignment.

We emphasize that the objective of this study is to explore whether physician agreement was influenced by characteristics of individual cases. As discussed elsewhere, greater agreement does not mean greater accuracy in cause of death assignment [32]. Therefore no inference can be made on the accuracy or external validity by studying physician agreement. However, we assumed that higher agreement should be correlated with better reliability of the physician coded cause of death. We also assumed that poor agreement is correlated with difficulty in classifying pregnancy-related deaths.

### Conclusion

To the best of our knowledge, there has been no other study which has examined physician agreement for pregnancy-related cause of death using verbal autopsies. This is the largest population-based study of inter-rater agreement of physician coded verbal autopsies, which provides greater precision to the statistical analysis, unavailable in the past to other pregnancy-related death studies.

Overall, we were reassured that there is substantial physician agreement and agreement is not significantly influenced by demographic or socio-economic factors or events related to the pregnancy, indicating that the tool will yield comparable results and is flexible in a variety of settings, and under a variety of conditions. As well, this paper informs ongoing physician coder training for the Million Death Study. In the absence of robust vital registration and universal healthcare services, verbal autopsies are an invaluable tool in providing proportional cause of death analysis for pregnancy-related deaths.

## Supporting Information

Figure S1 **Physician-coded flow diagram of million death study pregnancy-related deaths.**
(EPS)

Web Appendix S1 2011 Verbal Autopsy coding guidelines for pregnancy-related deaths.
(DOC)
