# Supplementary material for: Factors Associated with Physician Agreement and Coding Choices of Cause of Death Using Verbal Autopsies for 1130 Maternal Deaths in India
Source: PLoS One. 2012 Mar 28;7(3):e33075. doi: 10.1371/journal.pone.0033075 (PMC3314652; doi:10.1371/journal.pone.0033075)
Supplement: Web Appendix S1 — 2011 Verbal Autopsy coding guidelines for pregnancy-related deaths. (DOC) [file pone.0033075.s002.doc]

**Webappendix 1: Maternal Deaths ICD-10 Classification for Million Death Study, 2011**

Maternal death is the death of a woman while pregnant or within 42 days of termination of pregnancy, irrespective of the duration and site of the pregnancy, from any cause related to or aggravated by the pregnancy or its management, but not from accidental or incidental causes.

A **direct maternal death** (e.g. O00-O95, A34, F53) that is the result of a complication of the pregnancy, delivery, or their management, and an **indirect maternal death** (e.g. O98 or O99, or more specifically ICD-10 codes A-N) that is a pregnancy-related death in a patient with a preexisting or newly developed health problems, exacerbated by the pregnancy. Accidental death or incidental causes are coded according to the specific areas of the ICD-10 coding (e.g. V, W, X, Y).

| **ICD-10 CODE** | **CAUSE OF DEATH** | **2010** | **2005** |
| --- | --- | --- | --- |
| **O00** | **Ectopic pregnancy** | Gestational age ≤6 mos (usually ≤4 mos), severe abdominal pain and sudden collapse  **AND** Report from informant of physician diagnosis of extra-uterine pregnancy |  |
| **O01** | **Hydatidiform mole** | Not a cause of death |
| **O02** | **Other abnormal products of conception** | Not a cause of death |
| **O03** | **Spontaneous abortion;** complicated by  sepsis, hemorrhage or unspecified | Gestational age ≤6 mos **AND** Recent history of spontaneous vaginal bleeding  **AND** Any of the following:   - Infection: fever or pelvic pain, foul-smelling discharge pv, chills & rigour, +/- jaundice ≥4d post-miscarriage - Hemorrhage: profuse bleeding - Unspecified   **NOTE** if termination ≥7 mos, see codes O72, O75, or O85 | **O03-O06** Gestational age ≤6 mos Abortion (termination before 28 weeks of pregnancy) in less than 42 days before  death **AND** Any of the following:  • Lower abdominal pain  • Excessive vaginal bleeding  • Abnormal vaginal discharge  • Fever till death |
| **O04** | **Therapeutic abortion;** complicated by  sepsis, hemorrhage or unspecified | Gestational age ≤6 mos **AND** Intentional termination of the pregnancy by aspiration abortion performed in a health facility or medical abortion on the prescription of a physician  **AND** Any of the following:   - Infection: fever or pelvic pain, foul-smelling discharge pv, chills & rigour, +/- jaundice ≥4d post-abortion - Hemorrhage: profuse bleeding - Unspecified |
| **O05** | **Other abortion** (formerly ‘illegal abortion’); complicated by sepsis, hemorrhage, unspecified | Gestational age ≤6 mos **AND** Intentional termination of the pregnancy by means outside the regulated health facility, unsafe abortion practices (e.g. traditional medicine, aspiration abortion outside a health facility)  **AND** Any of the following:   - Infection: fever or pelvic pain, foul-smelling discharge pv, chills & rigour, or jaundice ≥4d post-abortion - Hemorrhage: profuse bleeding - Unspecified   **NOTE** if termination ≥7 mos, see codes O72, O75, or O85 |
| **O06** | **Unspecified abortion** (for early pregnancy termination undefined);complicated by sepsis, hemorrhage, unspecified | Gestational age ≤6 mos **AND** Termination of pregnancy by unspecified means, unclear whether spontaneous, therapeutic or other abortion  **AND** Any of the following:   - Infection: fever or pelvic pain, foul-smelling discharge pv, chills & rigour, +/- jaundice ≥4d post-abortion - Hemorrhage: profuse bleeding - Unspecified   **NOTE** if termination ≥7 mos, see codes O72, O75, or O85 |
| **O07** | **Failed attempted abortion** | Gestational age ≤6 mos **AND** Intentional termination of the pregnancy by aspiration abortion performed in a health facility or medical abortion on the prescription of a physician with report of referral for incomplete abortion.  **AND** Any of the following:   - Infection: fever or pelvic pain, foul-smelling discharge pv, chills & rigour, +/- jaundice ≥4d post-abortion - Hemorrhage: profuse bleeding - Unspecified |
| **O08** | **Complications following abortion and ectopic and molar pregnancy** | Not a cause of death |  |
| **O10-O16** | **Hypertension disorders of pregnancy (HDP)** | Gestational age ≥5 mos and <72h postpartum **AND** no fever, diarrhea and no h/o convulsions outside pregnancy  **AND** Any of the following:   - report of diagnosis of hypertension, either prior to or during the pregnancy - coma >1h duration - h/o fits in pregnancy, labour and/or postpartum - jaundice without fever in third trimester - signs and symptoms of HDP: upper right quadrant abdominal pain, severe frontal headache, blurred vision, generalized edema (not just ankle swelling)   **Possibly with**   - Gravida 1 - multiple gestation - intrauterine death diagnosed or stillbirth delivered - h/o of PPH - if ≥72h postpartum, consider O87 | **O15** History of convulsions for first time in pregnancy **OR** Doctors report of very high blood pressure with convulsions **Possibly with** Ankle swelling |
| **O20** | **Hemorrhage in early pregnancy** | Gestational age ≤6 mos **AND** profuse bleeding pv |  |
| **O21** | **Hyperemesis gravidarum** | Pregnant 1-10 mos **AND** report of excessive vomiting usually commencing <3mos continuing until death (not a new onset in late pregnancy) **AND** excessive weight loss & no signs of infection (fever, diarrhea) **AND** decreased muscle coordination, involuntary eye movement and dementia. |  |
| **O22** | **Venous complication in pregnancy**  Cerebrovenous sinus thrombosis | Gestational age 3-10 mos **AND** symptoms preceded by sudden onset of severe headache, possibly followed by vomiting, generalized seizures, confusion, sudden death **May have** h/o advanced maternal age, hypertension, |  |
| **O23** | **Infections of the genito-urinary tract in pregnancy** | Gestational age 1-10 mos **AND** fever **AND** report of diagnosis of pyelonephritis leading to septic shock **Possibly with** flank pain, abdominal/pelvic pain, nausea and vomitting |  |
| **O24** | **Diabetes mellitus in pregnancy** | Not a cause of death – see complications from pre-existing diabetes |  |
| **O25** | **Malnutrition** | Not a cause of death |  |
| **O26** | **Maternal care for other conditions predominantly related to pregnancy** | Gestational age 1-10 mos **AND**  unknown cause-of-death in pregnancy related condition unspecified though probably obstetric related  **Possibly with** previously healthy woman with no sign of other infection |  |
| **O28** | **Abnormal findings on antenatal screening** | Not a cause of death |  |
| **O29** | **Complication of anaesthesia during pregnancy** | Gestational age 1-10 mos **AND** report of anesthetic complication in surgery |  |
| **O30-O40** | **Maternal care** | Not a cause of death |  |
| **O41** | **Intra-amniotic infection /Endometritis** | Gestational age ≥6 mos **AND** New onset of fever around the time of labour **AND** Woman dies in labour or within 24h of delivery  **Possibly with**   - foul-smelling fluid leaking pv - h/o ruptured membranes for >24h - intrauterine death/stillbirth/neonatal death - increasing uterine pain not consistent with labour - preterm labour |  |
| **O42-O43** | **Premature rupture of membranes and placental disorders** | Not a cause of death |  |
| **O44** | **Placenta previa** | Gestational age ≥7 mos **AND** Maternal death due to hemorrhage *prior* to delivery **AND** No significant pain reported other than labour  **Possibly with** H/o painless episodes of bleeding often starting at 7 mos |  |
| **O45** | **Placental abruption** | Gestational age ≥7 mos **AND** Abdominal pain described as extreme, constant, abdomen is board-like  **Possibly with** H/o hypertension, trauma (accident, violence), bleeding pv (though can have concealed abruption), if delivery occurs - baby is stillbirth |  |
| **O46** | **Antepartum hemorrhage** | Gestational age ≥7 mos **AND** Woman dies *prior* to delivery **AND** profuse bleeding pv  **Possibly with** intrauterine death diagnosed prior to maternal death | Acute excessive bleeding in pregnancy after 28 weeks of gestation but before  birth of baby |
| **O47-O48** | **False labour and prolonged pregnancy** | Not cause of death |  |
| **O60-O66** | **Labour and obstructed labour** | Not a cause of death | **O64-O66** Abnormal presentation (breech, shoulder, hand or transverse) **AND** Baby notdelivered OR Difficulty in delivering baby, Forceps / vacuum delivery **AND**  Prolonged labour > 24 hours Prim.> 12 hr |
| **O67** | **Intrapartum hemorrhage** | Gestational age ≥7 mos **AND** Woman dies *during* delivery **AND** profuse bleeding pv | **O67** Excessive bleeding after delivery of baby, for example, blood completely  covering the floor or used many garments to soak blood |
| **O68-O70** |  | Not a cause of death |  |
| **O71** | **Other obstetric trauma**: uterine rupture in labour | Gestational age was ≥7 mos **AND** Woman dies undelivered or sudden collapse following delivery **AND** Any of the following:   - h/o prolonged (obstructed) labour - use of oxytocin induction/augmentation (‘injections to increase the pain’) - report of fundal pressure used during delivery - report of fetal malposition, i.e. transverse lie - h/o previous cesarean |  |
| **O71** | **Other obstetric trauma:** postpartum uterine inversion | Gestational age was ≥7 mos **AND** Uterus inverts with attempted delivery of placenta **Possibly with** uterus is not replaced by SBA |  |
| **O72** | **Postpartum hemorrhage** | Gestational age was ≥7 mos **AND** Woman dies *following* delivery of baby (can occur with placenta *in situ* or postpartum up to 14 days)  **AND** Any of the following:   - bleeding pv - if placenta undelivered, can have concealed bleeding and abdomen rises up as uterus fills with blood   **Possibly with** obstructed labour, signs and symptoms of infection in labour or postpartum, cesarean delivery, multiple gestation | **O72** Excessive bleeding after delivery of baby, for example, blood completely  covering the floor or used many garments to soak blood |
| **O73** | **Retained placenta, without hemorrhage** | Not a cause of death |  |
| **O74** | **Complications of anesthetic during labour and delivery** | Gestational age ≥7 mos **AND** Woman dies *during* delivery **AND** report of anesthetic complication in surgery |  |
| **O75** | **Complications of L&D** | Gestational age was ≥7 mos **AND** Woman dies in labour or within 24h of delivery, of probable direct maternal cause of death  **Possibly with**   - Previously healthy outside of pregnancy - May have developed complications during the pregnancy/delivery/postpartum |  |
| **O80-O84** | **Single and multiple delivery** | Not a cause of death |  |
| **O85** | **Puerperal sepsis** | Gestational age was ≥7 mos **AND** Death occurs ≥24h - ≤42d postpartum (usually day 3-14) **AND** fever or chills **OR** abdominal pain **OR** foul-smelling discharge pv  **Possibly with**   - jaundice appearing ≥4d postpartum - no h/o fever in pregnancy - h/o prolonged labour or ruptured membranes (>24h prior to delivery) - h/o preterm labour - h/o stillbirth/neonatal death - sweating/rigours/dizziness/headaches | High fever persisting till death **AND** Any of the following:  • Foul smelling vaginal discharge with or without blood  • Lower abdominal pain/distention  • Vomiting  **AND**  No cough, no burning; micturition; no yellowness of eyes |
| **O86** | **Other puerperal infectons:** cesarean wound, perineal repair | Gestational age was ≥7 mos **AND** Death occurs ≥24h - ≤42d postpartum **AND** Fever or chills **AND** H/o wound repair with descriptors of wound infection |  |
| **O87** | **Venous complications in the peurperium** | Gestational age ≥7 mos **AND** Death occurs ≤42d postpartum **AND** symptoms preceded by sudden onset of severe headache, possibly followed by vomiting, generalized seizures, confusion **May have** h/o advanced maternal age, hypertension, cesarean delivery (consider for eclamptic cases presenting ≥72h postpartum) |  |
| **O88** | **Obstetric embolism** | Gestational age was ≥7 mos **AND** sudden collapse, tachypnea, tachycardia, and hypoxemia  **Possibly with** h/o cardiac disease, DVT, delivery <8mos, multiple gestation (if following TA, code O08) |  |
| **O89** | **Complication of anaesthesia in the peurperium** | Postpartum post-delivery or abortion ≥24h - ≤42d **AND** report of anesthetic complication in surgery |  |
| **O90** | **Complications of the puerperal**  unspecified | Gestational age was ≥7 mos **AND** Death occurs ≥24h - ≤42d postpartum **AND** Woman dies in the postpartum period of probable direct maternal cause of death  **Possibly with**   - previously healthy outside of pregnancy - may have developed complications during the pregnancy/delivery/postpartum |  |
| **O91** | **Infection of the breast associated with childbirth** | Postpartum post-delivery ≥24h - ≤42d **AND** report of severe breast infection leading to sepsis | Postpartum ≥24h - ≤42d |
| **O92** | **Disorder of breasts** | Not a cause of death |  |
| **O94** | **Sequelae of complication** | Not a cause of death |  |
| **O95** | **Obstetric death** of unspecified cause, during the pregnancy, L&D or postpartum | Death of a woman from gestational age 1-10 mos to 42 days postpartum/postabortion **AND** Most probably a direct maternal cause of death  **NOTE** Use O75 or O90 if clearly in the intrapartum period or postpartum period respectively. O95 can be used for sudden death not related to O98 or O99 |  |
| **O96** | **Late maternal death** | Death of woman between *43-365 days postpartum* of most probably a direct maternal cause of death  **Possibly with**   - previously healthy outside of pregnancy - may have developed complications during the pregnancy/delivery/postpartum - report of near-miss event^1^ in pregnancy/delivery/postpartum period |  |
| **O97** | **Death from sequalae of direct obstetric causes** | Death of woman *>366 days postpartum* of most probably a direct maternal cause of death  **Possibly with**   - previously healthy outside of pregnancy - may have developed complications during the pregnancy/delivery/postpartum - report of near-miss event^1^ in pregnancy,/delivery/postpartum period |  |
| **O98** | **Maternal infectious and parasitic diseases classifiable elsewhere but complicating pregnancy, childbirth and the puerperium** | Death of a woman who is pregnant/intrapartum/postpartum (≤42 days) of probable infectious cause of death – possibly exacerbated by pregnancy  **Possibly with:**   - h/o of fever prior to pregnancy, or prior to puerperium - h/o intermittent fever - jaundice +/- fever (not postpartum/postabortion, no signs and symptoms of HDP) - diarrhea   Consider a more specific ICD code if possible |  |
| **O99** | **Other maternal diseases classifiable elsewhere but complicating pregnancy, childbirth and the puerperium** | Death of a woman who is pregnant/intrapartum/postpartum (≤42 days) of probably non-infectious cause of death, possibly exacerbated by the pregnancy  **Possibly with**   - h/o pre-existing medical condition - reported diagnosis of new medical condition   Consider a more specific ICD code if possible |  |
| **A34** | **Obstetric tetanus** | Maternal death; postabortion or postpartum **AND** Reports unable to swallow or neck stiffness **AND** Rigid spasms  **Possibly with**   - incubation 4-42 days, average 12 days, though may be earlier if h/o prolonged labour reported - no ANC attendance, no report of TT injection in pregnancy - *risus sardonicus* - febrile convulsions - episodes of cyanosis (“she became black”) |  |
| **F53** | **Mental and behavioural disorders associated with the peurperium** | Postpartum suicide with signs and symptoms of postpartum depression or psychosis |  |

^1^any pregnant or recently delivered woman, in whom immediate survival was threatened and who survives by chance or due to the hospital care she received

Abbreviations: AFE – amniotic fluid embolism, ANC - antenatal care, APH - antepartum hemorrhage, DVT – deep vein thrombosis, h – hour, h/o – history of, HDP – hypertensive, L&D – labour and delivery, mos – months, PE – pulmonary embolism, PPH – postpartum hemorrhage, pv – per vaginum, SBA – skilled birth attendant, TA - therapeutic abortion, TB – tuberculosis, TT – tetanus toxoid vaccine
